# Cannabis Use During Pregnancy Correlates With Adverse Maternal Mental Health Outcomes: A Retrospective Study

**DOI:** 10.7759/cureus.82146

**Published:** 2025-04-12

**Authors:** Kypros J Dereschuk, Eduardo Espiridion

**Affiliations:** 1 Psychiatry, Drexel University College of Medicine, Philadelphia, USA; 2 Psychiatry, Tower Health Medical Group, West Reading, USA

**Keywords:** adverse pregnancy outcome, alcohol misuse, cannabis use, maternal depression, maternal mental health, panic disorder (pd), suicidal ideation

## Abstract

Introduction

The prevalence of cannabis use during pregnancy has risen alongside its legalization and perceived safety, often being used to alleviate pregnancy-related discomforts. However, cannabis use during pregnancy may have adverse implications for maternal mental health, including increased rates of depression, panic disorder, suicidal ideation, and alcohol abuse. This study aims to evaluate the association between cannabis use during pregnancy and these mental health outcomes.

Methods

This retrospective cohort study utilized the TriNetX database, including over 2 million pregnant patients from 69 U.S. healthcare organizations (HCOs). A cohort of 51,087 cannabis users during pregnancy was compared to 1,936,508 non-users. Outcomes analyzed included depression, panic disorder, suicidal ideation, and alcohol abuse, identified using the International Classification of Diseases, 10th Revision (ICD-10) codes. Risk ratios, hazard ratios, and Kaplan-Meier survival probabilities were calculated, with statistical significance set at p < 0.05.

Results

Cannabis use during pregnancy was associated with higher incidences of all four mental health outcomes. Alcohol abuse showed the greatest relative risk (risk ratio = 13.57; hazard ratio = 12.44), followed by suicidal ideation (risk ratio = 10.67; hazard ratio = 9.81), panic disorder (risk ratio = 5.47; hazard ratio = 5.01), and depression (risk ratio = 2.66; hazard ratio = 3.50). Depression affected 29.7% of cannabis users, compared to 11.2% of non-users, with significant differences in survival probabilities (p < 0.001).

Conclusion

Cannabis use during pregnancy is significantly associated with increased risks of adverse mental health outcomes. These findings emphasize the importance of screening for cannabis use and mental health conditions during pregnancy and underscore the need for public health initiatives addressing the risks of prenatal cannabis use. Further research is needed to explore causal relationships and dosing effects.

## Introduction

The prevalence of cannabis use during pregnancy has risen in recent years, in part due to the growing legalization and perception of cannabis as a relatively safe substance [1]. Pregnant individuals may use cannabis to alleviate nausea, anxiety, or other discomforts associated with pregnancy, often without fully understanding the potential risks involved [2]. However, emerging evidence suggests that cannabis use during pregnancy may have significant implications for both maternal and fetal health, including potential associations with adverse mental health outcomes for the pregnant individual; furthermore, national guidelines advise abstinence from cannabis in pregnant individuals [3,4].

Mental health disorders, such as major depressive disorder (MDD), suicidal ideation, panic episodes, and alcohol use disorder, are critical concerns during pregnancy given their impact on maternal well-being and, subsequently, childhood outcomes [5]. Additionally, substance use during pregnancy, including alcohol abuse, has been shown to pose a risk not only to the pregnant mother’s health but also to neonatal outcomes, such as preterm birth, small size for gestational age, and developmental delays [6,7].

Maternal cannabis use has been associated with various adverse physical health effects, including an increased risk of hypertension and preeclampsia [8]. Despite the growing body of literature on the consequences of cannabis use in the general population, relatively few studies have examined its specific impact on the mental health of pregnant individuals. A recent study by Andrade observed an association between cannabis use during pregnancy and the likelihood of maternal health conditions [9]. While Constantino-Pettit et al. found that cannabis use across the prenatal period did not associate with a more significant decline in stress or depression symptoms, compared to those who did not use cannabis [10].

The interplay between cannabis use during pregnancy and mental health outcomes remains underexplored, particularly regarding its association with disorders such as MDD, panic disorder, alcohol abuse, and suicidal ideation. To address this gap, our study explores the relationship between the mental health outcomes of pregnant individuals who used cannabis during their pregnancy and those who did not. Specifically, we investigated the odds of developing MDD, panic disorder, experiencing suicidal ideation, and engaging in alcohol abuse.

## Materials and methods

This retrospective cohort study was conducted using data from TriNetX, a global federated collaborative health research network spanning over 118 million patient records, with comprehensive longitudinal data from 69 healthcare organization (HCO) systems in the U.S. alone [11]. TriNetX offers research access to extensive data that is de-identified, continuously updated, and provides a variety of data points, including labs, diagnoses, medications, procedures, and demographics. Additionally, it contains 44 million tokenized patients, linkable to third-party datasets. The database is designed to ensure data accuracy and completeness through rigorous quality control processes and standardized data extraction methods across participating HCOs. The completeness of the database is robust, including the entire patient population at every partnering HCO. It is important to note that the TriNetX data is not publicly accessible. For this study, data from TriNetX was accessed via connection through Drexel University and limited to the U.S., spanning 69 HCOs and 118 million patient records from 2004 to 2024.

We identified two cohorts utilizing inclusion and exclusion criteria from the International Classification of Diseases, 10th Revision (ICD-10) codes. Our study compared four outcomes between the two cohorts. The control cohort was defined as having the procedure code “UMLS:ICD10PCS:10 Pregnancy”. This query produced our control cohort with 1,936,508 patients. The comparison cohort, which we named the cannabis use in pregnancy (CUP) cohort, was defined with the following criteria: age between 13 and 55 years (most recent occurrence of pregnancy used), demographic code “UMLS:HL7V3.0:Gender:F Female”, diagnosis code “UMLS:ICD10CM:F12.9 Cannabis use, unspecified”, diagnosis code “UMLS:ICD10CM:F12.90 Cannabis use, unspecified, uncomplicated”, and procedure code “UMLS:ICD10PCS:10 Pregnancy”. This query produced our CUP cohort with 51,087 patients.

For this study, our four outcomes of interest were defined as follows. Depression was defined as “UMLS:ICD10CM:F32 Depressive episode”. Panic disorder was defined as “UMLS:ICD10CM:F41.0 Panic disorder (episodic paroxysmal anxiety)”. Suicidal ideations were defined as “UMLS:ICD10CM:R45.851 Suicidal ideations”. Alcohol abuse was defined as “UMLS:ICD10CM:F10.1 Alcohol abuse”. As TriNetX data is deidentified, IRB approval was not required for our study. The data was queried using the ICD-10 codes because the TriNetX platform employs this method for querying the electronic health record (EHR).

In the two-comparison cohort component of the study, measures of association and survival were assessed using the TriNetX platform. Risk difference and risk ratio were calculated to compare cohorts. Survival was extrapolated using a Kaplan-Meier survival analysis, followed by a log-rank test and Cox hazard ratio and proportionality test. The survival probability of the observed outcome at the end of the time window was calculated and compared. In this study, survival probability refers to the probability that a patient survives without experiencing one of the four outcomes (depression, panic disorder, suicidal ideation, or alcohol abuse), so a higher proportion would indicate fewer patients in that population experienced the respective adverse mental health outcome. The log-rank test was used to assess significance (p < 0.05). Hazard ratios were calculated using the Cox proportional hazards model. The index event was defined using ICD-10 codes. For the CUP cohort, the index event was defined by the following criteria: diagnosis code “UMLS:ICD10CM:F12.9 Cannabis use, unspecified” and diagnosis code “UMLS:ICD10CM:F12.90 Cannabis use, unspecified, uncomplicated” and procedure code “UMLS:ICD10PCS:10 Pregnancy”. The index event for the control cohort was defined as the procedure code “UMLS:ICD10PCS:10 Pregnancy”. This analysis included outcomes that occurred in the time window that started one day after the first occurrence of the index event.

## Results

Utilizing the U.S. collaborative network on TriNetX, we identified 51,087 patients in the CUP group and 1,936,508 patients in the control group. Demographics were comparable between both cohorts (Table 1). The CUP group (n = 51,087) had an average age at the time of pregnancy of 27.4 years (SD ± 6.27), while the control group (n = 1,936,508) had an average age of 29.3 years (SD ± 6.51). In terms of gender, 100% of the CUP group were female, whereas, in the control group, 98% (n = 1,891,928) were female, less than 1% (n = 4,857) identified as male, and 2% (n = 39,723) were of unknown gender. The reported races of the control group were 54% (n = 1,047,000) White individuals, 15% (n = 292,599) Black or African American individuals, 7% (n = 126,509) Asian individuals, less than 1% (n = 6,029) American Indian or Alaska Native individuals, 1% (n = 10,802) Native Hawaiian or other Pacific Islander individuals, 7% (n = 140,097) categorized as other, and 16% (n = 313,472) categorized as unknown. The reported races of the CUP group were 53% (n = 26,986) White individuals, 32% (n = 16,197) Black or African American individuals, 1% (n = 263) Asian individuals, 1% (n = 369) American Indian or Alaska Native individuals, less than 1% (n = 205) Native Hawaiian or other Pacific Islander individuals, 5% (n = 2,664) categorized as other, and 9% (n = 4,403) categorized as unknown. With regard to ethnicity, 17% (n = 338,676) of the control group identified as Hispanic or Latino, 58% (n = 1,123,934) identified as not Hispanic or Latino, and 24% (n = 473,898) were of unknown ethnicity, whereas, in the CUP group, 11% (n = 5,531) identified as Hispanic or Latino, 75% (n = 38,066) identified as not Hispanic or Latino, and 15% (n = 7,490) had unknown ethnicity.

**Table 1:** Demographics

	Control Group (n = 1,936,508)	Cannabis Use Group (n = 51,087)	p-value
Age, Mean ± SD	29.3 ± 6.51	27.4 ± 6.27	<0.0001
Gender			
Female, n (%)	1,891,928 (98%)	51,087 (100%)	<0.0001
Male, n (%)	4,857 (0%)	0 (0%)	<0.0001
Unknown, n (%)	39,723 (2%)	0 (0%)	<0.0001
Race			
White Individuals	1,047,000 (54%)	26,986 (53%)	<0.0001
Black or African American Individuals	292,599 (15%)	16,197 (32%)	<0.0001
Asian Individuals	126,509 (7%)	263 (1%)	<0.0001
American Indian or Alaska Native Individuals	6,029 (0%)	369 (1%)	<0.0001
Native Hawaiian or Other Pacific Islander Individuals	10,802 (1%)	205 (0%)	<0.0001
Other	140,097 (7%)	2,664 (5%)	<0.0001
Unknown, n (%)	313,472 (16%)	4,403 (9%)	<0.0001
Ethnicity			
Hispanic or Latino	338,676 (17%)	5,531 (11%)	<0.0001
Not Hispanic or Latino	1,123,934 (58%)	38,066 (75%)	<0.0001
Unkown, n (%)	473,898 (24%)	7,490 (15%)	<0.0001

In this analysis, comparing mental health outcomes between pregnant women with documented cannabis use during their pregnancy to those without cannabis use, all examined conditions showed higher risk and odds in the CUP cohort (Table 2). Of the 51,087 patients in the CUP group, 1,109 had alcohol abuse, 1,240 had suicidal ideations, 1,077 had panic disorder, and 15,175 had a depressive episode, for risks of 0.022, 0.024, 0.021, and 0.297, respectively. Of the 1,936,508 patients in the control group, 3,098 had alcohol abuse, 4,404 had suicidal ideations, 7,464 had panic disorder, and 216,356 had a depressive episode, for risks of 0.002, 0.002, 0.004, and 0.112, respectively. The odds ratio and risk ratio both reflect this result, with the risk ratio being 13.569 (95% CI = 12.677-14.524) for alcohol abuse, 10.673 (95% CI = 10.027, 11.360) for suicidal ideations, 5.470 (95% CI = 5.134, 5.827) for panic disorder, and 2.659 (95% CI = 2.622, 2.696) for depressive episode. The odds ratio was 13.854 (95% CI = 12.928, 14.846) for alcohol abuse, 10.914 (95% CI = 10.241, 11.631) for suicidal ideations, 5.566 (95% CI = 5.218, 5.937) for panic disorder, and 3.360 (95% CI = 3.295, 3.426) for a depressive episode.

**Table 2:** Measures of association: cohort statistics, risk ratio, and odds ratio

Mental Health Outcome	Cannabis Use During Pregnancy Risk	No Cannabis Use Risk	Risk Ratio (95% CI)	Odds Ratio (95% CI)
Alcohol Abuse	0.022 (1,109/51,087)	0.002 (3,098/1,936,508)	13.569 (12.677, 14.524)	13.854 (12.928, 14.846)
Suicidal Ideations	0.024 (1,240/51,087)	0.002 (4,404/1,936,508)	10.673 (10.027, 11.360)	10.914 (10.241, 11.631)
Panic Disorder	0.021 (1,077/51,087)	0.004 (7,464/1,936,508)	5.470 (5.134, 5.827)	5.566 (5.218, 5.937)
Depressive Episode	0.297 (15,175/51,087)	0.112 (216,356/1,936,508)	2.659 (2.622, 2.696)	3.360 (3.295, 3.426)

The difference in risk was significant across all four outcomes, utilizing z-tests. For alcohol abuse, z = 97.616, p < 0.001, 95% CI = (0.019, 0.022). For suicidal ideations, z = 92.230, p < 0.001, 95% CI = (0.021, 0.023). For panic disorder, z = 58.758, p < 0.001, 95% CI = (0.016, 0.018). For depressive episode, z = 128.876, p < 0.001, 95% CI = (0.181, 0.189) (Table 3).

**Table 3:** Measures of association: risk difference

Mental Health Outcome	Risk Difference	95% CI Lower	95% CI Upper	z	p-value
Alcohol Abuse	0.021	0.019	0.022	97.616	<0.001
Suicidal Ideations	0.022	0.021	0.023	92.230	<0.001
Panic Disorder	0.017	0.016	0.018	58.758	<0.001
Depressive Episode	0.185	0.181	0.189	128.876	<0.001

**Figure 1:**
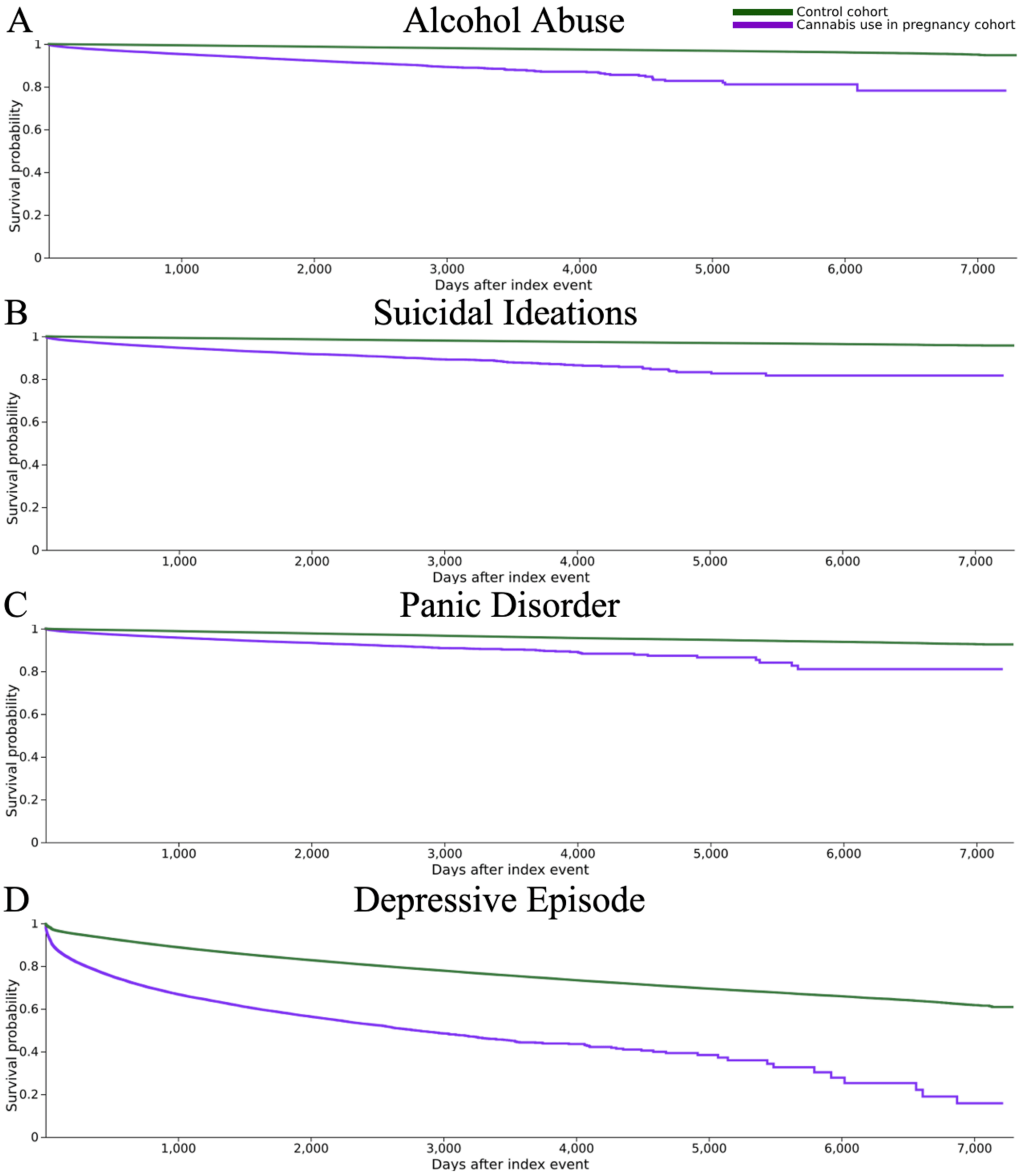
Kaplan-Meier analysis: survival curves Kaplan-Meier survival curves for each specified mental health outcome (A) alcohol abuse; (B) suicidal ideation; (C) panic disorder; (D) depressive episode. The index event is pregnancy.

The expected survival and hazard ratios were calculated via a Kaplan-Meier analysis. For the CUP group, survival probabilities were 78.19% for alcohol abuse, 81.62% for suicidal ideations, 81.06% for panic disorder, and 15.78% for depressive episode. For the control group, survival probabilities were 94.74% for alcohol abuse, 95.71% for suicidal ideations, 92.57% for panic disorder, and 60.85% for depressive episode (Figure 1).

Log-rank tests proved this difference to be statistically significant, with the control group having a significantly higher expected survival rate for all four outcomes compared to the CUP group. For alcohol abuse, χ² = 12,021.425, p < 0.001; for suicidal ideations, χ² = 12,433.472, p < 0.001; for panic disorder, χ² = 3,270.238, p < 0.001; and for depressive episode, χ² = 25,459.193, p < 0.001 (Table 4).

**Table 4:** Kaplan-Meier analysis: log-rank test χ^2^: Chi-square; df: degrees of freedom

Mental Health Outcome	χ^2^	df	p-value
Alcohol Abuse	12,021.425	1	<0.001
Suicidal Ideations	12,433.472	1	<0.001
Panic Disorder	3,270.238	1	<0.001
Depressive Episode	25,459.193	1	<0.001

Finally, Kaplan-Meier analysis, utilizing proportionality tests, found the CUP group to be statistically significantly more likely to experience each of the four outcomes. For alcohol abuse, hazard ratio = 7.054, χ² = 223.959, p < 0.001; for suicidal ideations, hazard ratio = 6.668, χ² = 189.606, p < 0.001; for panic disorder, hazard ratio = 3.293, χ² = 117.022, p < 0.001; and for depressive episode, hazard ratio = 3.301, χ² = 394.081, p < 0.001 (Tables 5-6).

**Table 5:** Kaplan-Meier analysis: hazard ratio

Mental Health Outcome	Hazard Ratio	95% CI Lower	95% CI Upper
Alcohol Abuse	7.054	6.773	7.347
Suicidal Ideations	6.668	6.416	6.931
Panic Disorder	3.293	3.154	3.439
Depressive Episode	3.301	3.250	3.353

**Table 6:** Kaplan-Meier analysis: proportionality χ^2^: Chi-square; df: degrees of freedom

Mental Health Outcome	χ^2^	df	p-value
Alcohol Abuse	223.959	1	<0.001
Suicidal Ideations	189.606	1	<0.001
Panic Disorder	117.022	1	<0.001
Depressive Episode	394.081	1	<0.001

## Discussion

While the study of substance use, such as cannabis, during pregnancy on fetal health has been a recent focus of research, the effects of cannabis use during pregnancy on maternal health - particularly maternal mental health - are relatively less well studied [12]. This study aimed to evaluate the relationship between cannabis use during pregnancy and four mental health outcomes: MDD, panic disorder, suicidal ideation, and alcohol abuse. After conducting a query across 69 HCOs and identifying a cohort of 51,087 patients who used cannabis during their pregnancy, and a cohort of 1,936,508 patients who did not, we observed a significantly higher rate of all four outcomes in the cohort that used cannabis. Most notable was the risk of alcohol abuse, which was over 13 times higher in cannabis users. Depression affected nearly one-third of cannabis-using mothers, compared to about one-tenth of non-users.

These findings build upon previous studies. For example, a cross-sectional study by Goodwin et al. found that pregnant individuals who used cannabis were significantly more likely to experience symptoms of depression and perceived stress, whereas a study by El Marroun et al. reported an association between maternal cannabis use and alcohol use, being single, childhood trauma, and delinquency; however, unlike our findings, they did not observe an association with psychopathology, including depression [13,14]. Our study expands upon this previous literature by examining, in addition to depression, panic disorder, suicidal ideation, and alcohol abuse in a large national cohort. Furthermore, our findings are consistent with those of Andrade, who found an association between cannabis use during pregnancy and maternal health conditions, and Constantino-Pettit et al., who did not observe significant reductions in stress or depression among prenatal cannabis users [9,10]. Previous studies have investigated the association of maternal cannabis use with a variety of neuropsychiatric disorders, including attention-deficit/hyperactivity disorder, autism spectrum disorder, depression, anxiety, and psychotic disorders [15]. To our knowledge, our study is among the first large-scale, multi-institutional cohort studies to report that cannabis use during pregnancy is associated with significantly increased risks of suicidal ideation and panic disorder. 

Our study contributes to the growing body of evidence on the potential harms of cannabis use, particularly during pregnancy. These findings support the importance of screening for cannabis use and mental health conditions during pregnancy. This knowledge may prove useful to healthcare providers by expanding their understanding of the relationship between prenatal cannabis use and maternal mental health outcomes. These findings also have implications for public health campaigns. Public health campaigns should address the risks to maternal mental health associated with cannabis use during pregnancy. This is particularly true in areas where cannabis use is legal and where it is perceived as low risk. This is becoming increasingly important, as the perception of cannabis use as low risk increased by 86% (from 16.8% to 31.2%) between 2002 and 2014, and by 19% (from 30.1% to 35.8%) between 2015 and 2018 [16]. Public health initiatives should prioritize education around the mental health risks of prenatal cannabis use, given these shifting perceptions of cannabis safety and increasing legalization.

A major strength of our study is the use of the TriNetX database, allowing for access to a large, diverse cohort of patients across multiple different HCOs. This increases the external validity of our study and the power of our statistical analyses. Furthermore, our focus on four well-defined mental health outcomes provides insight into multiple potential psychological risks associated with cannabis use during pregnancy and reduces the risk of misclassification bias.

Our study also has several limitations. The observational design precludes any inference of causality between prenatal cannabis use and our four mental health outcomes. Thus, it remains unclear from our study whether cannabis use contributes to the development of these mental health outcomes, whether the presence of these mental health outcomes contributes to prenatal cannabis use, or whether both are influenced by confounding factors. Further, the nature of our study, which retrospectively analyzes chart data, relies on accurate documentation and limits our ability to control for potential confounders. Additionally, cannabis use was identified using ICD-10 codes documented in the EHR. This method is subject to underreporting and potential misclassification, as it relies on accurate patient disclosure and clinician documentation. As such, some individuals who used cannabis during pregnancy may not have been captured, potentially biasing our findings. Furthermore, social stigma regarding cannabis use may also lead to underreporting or misclassification of cannabis use [17]. Confounding by variables such as socioeconomic status, poly-substance use, and prior mental health history is a further limitation. These factors may influence both cannabis use during pregnancy and mental health outcomes, thus influencing our observed associations. Lastly, our study does not assess the impact of different doses of cannabis on the mental health outcomes.

Future studies should aim to address the limitations in our study. Prospective cohort studies with greater detail regarding cannabis dosing, frequency, and method of administration will offer further insight into the potential impact of prenatal cannabis use on maternal mental health. Utilizing robust controls for confounding variables will also aid in inferring causality between cannabis use during pregnancy and maternal mental health outcomes.

## Conclusions

Our large retrospective cohort study provides evidence of a significant association between cannabis use during pregnancy and increased rates of MDD, panic disorder, alcohol abuse, and suicidal ideation. These findings highlight the importance of heightened clinical awareness about prenatal cannabis use and mental health conditions in pregnant individuals, particularly as cannabis becomes more widely legalized and perceived as a low-risk substance. These results also emphasize the importance of increased cannabis use screening during pregnancy to mitigate potential risks associated with cannabis use. Public health initiatives should prioritize education around the mental health risks of prenatal cannabis use, especially given increasing legalization and shifting perceptions of cannabis safety. Given the limitations of our study, including its retrospective design and the potential for confounding variables, future research should focus on prospective studies that account for cannabis dosage, frequency, and method of administration to better understand the potential causal relationship between prenatal cannabis use and maternal mental health outcomes.
